# The breath and mind connection in young people with post-COVID syndrome: feasibility and acceptability of a pilot randomised co-designed intervention

**DOI:** 10.1007/s00431-026-06840-7

**Published:** 2026-03-16

**Authors:** Charlotte Wells, Deborah Christie, Rebecca Johnston, Faye Knight, Monica Samuel, Terry Y. Segal, Mark Shevlin, Rachel Sparrow, Deborah Woodman, Samatha Sonnappa

**Affiliations:** 1 University of Suffolk, Ipswich, UK; 2 Department of Paediatric and Adolescent Medicine, University College London Hospital NHS Foundation Trust, London, UK; 3 Guys and St Thomas’s NHS Foundation Trust, London, UK; 4 Department of Psychology, Ulster University, Coleraine, Northern Ireland; 5 Department of Respiratory Paediatrics, The Royal Brompton Hospital part of Guy’s and ST Thomas’s NHS Foundation Trust, London, UK

**Keywords:** Young people, Narrative therapy, Post-COVID syndrome, Breathing retraining

## Abstract

Post-COVID syndrome is widely recognised in children and young people (CYP), where one or more persistent physical symptoms are present 12 weeks after the initial infection that cannot be explained by an alternative cause. We aimed to assess whether an intervention based on narrative therapy and breathing pattern retraining, co-designed with CYP with PCS (12–18 years) was feasible and acceptable within an NHS service. Participants were randomised to standard clinical care or intervention plus standard clinical care. The primary aim was to assess acceptability and feasibility within the study cohort. Intervention effects were evaluated using the Strengths and Difficulties Questionnaire (SDQ) Impact Score,with additional secondary outcomes included the Nijmegen questionnaire, Chandler Fatigue, and EQ-5D-Y, wellness rating, pulmonary function, and exercise testing. Thirty-two CYP were randomised to standard treatment (*n* = 15) or intervention (*n* = 17), mean age of 15 years, and 11 (34%) male. Twenty-seven (84%) were diagnosed with breathing pattern disorders. Four intervention groups were offered over 6 months. 14/17 completed the session. Qualitative feedback was positive. There was no statistically significant improvement in the SDQ over time or between groups. Nearly all secondary outcome measures showed statistically significant improvement over time (< 0.001) but not between groups. *Conclusions*: The intervention was acceptable and feasible to run in an NHS clinic setting for CYP with post-COVID syndrome. Significant improvements seen with time in secondary outcome measures in both groups indicate that post-COVID symptoms improve with time independent of the intervention.

**Table:** 

**What is known – What is new:** • *Post-COVID syndrome (PCS) affects up to 66.5% of children and young people (CYP) with common persistent or fluctuating symptoms of fatigue, headaches, anxiety, brain fog, breathlessness, and post-exertional malaise and symptom exacerbation.* • *Few interventions have sought to address both the physical and mental health impact of PCS in adults and the evidence remains limited for CYP.* • *This novel psychology/physiotherapy co-designed intervention is acceptable and feasible in an NHS clinic setting.* • *We identified that breathing pattern disorders are common and a predominant cause of breathlessness in teenagers with PCS.*

**Supplementary Information:**

The online version contains supplementary material available at 10.1007/s00431-026-06840-7.

## Introduction

Post-COVID syndrome (PCS), also known as “long COVID” or post COVID condition, presents as persistent, sometimes fluctuating symptoms developed following a SARS-CoV-2 infection interfering with previous functional ability. PCS is seen in 2–8% of children and young people (CYP) who had a history of confirmed SARS-CoV-2 infection [1, 2]. Nearly half of CYP with PCS report experiencing breathlessness and over half report anxiety and low mood [3]. These problems may be interrelated, exacerbate each other, and cause significant morbidity in terms of missing school, social and other activities affecting adolescent biopsychosocial development.

After exclusion of alternative diagnoses, the key principle of care for PCS is holistic multidisciplinary team (MDT) support and rehabilitation. PCS management guidelines are based largely on expert opinion, advocating personalised holistic approaches to support recovery [3]. Treatment strategies include medical treatment of comorbidities such as migraine or dysautonomia, patient information leaflets, online psychoeducation webinars, and access to individualised therapy from specialist physiotherapists, occupational therapists, clinical psychologists, psychotherapists, psychiatrists, dieticians, and clinical nurse specialists. A regional PCS MDT was established across London in 2020 to tailor assessment, management, and rehabilitation support for CYP in collaboration with local referrers.

A key research priority is addressing the absence of evidence-based interventions in this clinical area given the limited data on the comorbidity of mental health effects and PCS in CYP. Chronic illness can leave CYP feeling disconnected from education, friends, and usual activities, impacting on emotional wellbeing. Nearly half of parents surveyed reported changes in their children’s mood since having COVID-19 [4].

Breathlessness (dyspnoea) is a common symptom of PCS [5], potentially linked to autonomic nervous system disturbances that cause breathing dysregulation. This dysregulation may stem from viral or immune-mediated disruption, leading to breathlessness, chest pain, palpitations, and orthostatic intolerance [6]. Chronic breathing pattern changes can result in dyspnoea even without respiratory disease [7, 8], due to biochemical, biomechanical, or psycho-physiological factors [9, 10]. Breathing retraining, guided relaxation, and mindfulness help alleviate symptoms and improve breathing pattern. Philip et al. [11] showed an online programme improved psychological health and breathlessness in adults with PCS. A one-year follow-up study [12] found dyspnoea (90%), fatigue (70%), and hyperventilation syndrome (HVS) (34%) were common in PCS patients, often associated with anxiety.

Complex reciprocal relationships between triggers, conditioning, and persistent PCS symptoms, like other functional disorders, require a holistic approach to assessment and management rather than being simply based on pathophysiological findings have been described [13]. Although the link between PCS, mental health, and dysfunctional breathing is described clinically, there are no published or ongoing studies jointly addressing these two related issues.

A novel package of online resources and a half-day telehealth psychology/physiotherapy intervention was co-designed with young people who had been through specialist PCS MDT services. *A manuscript describing the co-design process is in preparation; the lead author can be contacted for further details*. This study aims to explore the acceptability and feasibility of delivering the intervention adapted from existing programmes [14, 15]. This includes a “journey of life” narrative therapy approach to connect patients to their strengths and experiences to improve wellbeing, as well as breathing exercises designed to improve breathing patterns. The study documented symptoms in this population and described standard treatment in two tertiary PCS clinics. The primary aim was to assess acceptability and feasibility by monitoring attendance rates and using both open-ended and forced-choice questionnaire items to elicit views on the intervention. Exploratory statistical analyses were conducted to evaluate changes in psychological and health-related variables between the control and treatment group.

## Design

This is a randomised pilot trial assessing acceptability and feasibility of a co-designed intervention.

### Participants

#### Inclusion criteria

1. Young people aged between 12–18 years attending the regional tertiary MDT PCS service from 1 st Jan to December 15th 2023, with a history of symptoms affecting activities of daily living for more than 3 months, not explained by another condition and evidence of suspected COVID-19 infection with one of three criteria:
  a Previous PCR positive for SARS-CoV-2
  b COVID antibody positivity
  c Clear epidemiological links determined on a case-by-case basis.
2. English of a standard adequate to participate in a group intervention

#### Exclusion criteria

The following are the exclusion criteria: significant mental health or neurodevelopmental difficulties, e.g. severe autism spectrum disorder, attention deficit hyperactivity disorder, intellectual disability, and/or high psychiatric risk, e.g. suicidality, severe emotional or behavioural dysregulation precluding participation in group intervention.

## Methods

Potential participants and their parent/guardian were introduced to the research project by their clinical team and given an age-appropriate information leaflet (young people aged 12 to 15, 16 to 18 or for adult parent/guardian). The lead researcher contacted those who expressed an interest to take part to gain informed assent for young people aged 12 to 15 or consent for young people aged 16 to 18. Parental consent was obtained for all CYP.

### Randomisation and blinding

CYP were assigned into 2 treatment groups using computer-generated block randomisation with variable block sizes to receive either standard treatment or standard treatment plus intervention. Participants were allocated after the initial assessment at which point participants (and the lead researcher, CW) were no longer blinded. Clinical teams and physiologists collecting timepoint two data were masked from group allocations.

### Ethics

Ethics for a two-part research project to co-design and then pilot a new intervention for CYP with PCS was approved by Yorkshire and the Humber–Leeds East Research Ethics Committee (IRAS number: 315063) in November 2022.

### Co-designed intervention

Four 3.5-h online group sessions were delivered over six months to the intervention group. Each participant attended one session. Each group was facilitated by a clinical specialist physiotherapist (CW), a consultant clinical psychologist (DC), and a clinical psychologist (RS). The group was based on a “journey of life” metaphor. This invites young people to connect to their strengths and tell their stories in ways that make them feel stronger (see supplementary materials). This narrative approach encourages richer narratives of life, rather than traditional, often disparate descriptions focusing on illness [16]. Additional breathing exercises were integrated into the “journey”.

Following the group, participants were sent the link to an evolving interactive digital resource which represented their story and gave links to breathing videos to continue their home practice: This is available to view at https://sway.cloud.microsoft/GFCmMaVbOFRhmzNA?ref=Link&loc=play.

### Baseline assessment

Demographic data and wellbeing scores are collected in clinics using the national International Severe Acute Respiratory and Emerging Infection Consortium (ISARIC) PCS survey when the referral to the tertiary service is accepted.

### Primary outcome measure

#### Strengths and difficulties questionnaire (SDQ) impact score

The Strengths and Difficulties Questionnaire (SDQ) Impact Score reflects the experienced impact of emotional symptoms, conduct problems, hyperactivity/inattention, and peer relationship problems. Scores range from 0 to 10 with 3–10 in the very high range.

### Secondary outcome measures

#### EQ-5D-Y

The EQ-5D-Y-3L reflects health-related quality of life for children and adolescents, covering mobility, self-care, usual activities, pain/discomfort, and anxiety/depression.

#### 11-item chandler fatigue questionnaire

The self-report 11-item Chalder Fatigue Questionnaire (CFQ-11) assesses physical and mental fatigue impacting on daily functioning over the past month. A score of 4 or higher is clinically significant.

#### Wellness score (0–100)

Overall health is rated on that specific day from 0 (worst health you can think of) to 100% (best health you can think of).

#### Nijmegen questionnaire

The Nijmegen questionnaire is part of a multidimensional assessment of breathing pattern disorders (BrPD) in adults [17, 18]. Although it has not been validated in children, it was used to describe reported symptoms in the participants [19, 20].

#### Pulmonary function assessments

Spirometry [21] and Cardiopulmonary Exercise Test (CPET) [22] were performed using standard guidelines and equations with Jaeger Vyntus CPX equipment. Body plethysmography and gas transfer were performed according to ATS/ERS standards [23]. For methodological details on CPET and BrPD diagnosis, see supplementary information.

#### Acceptability and feasibility

This was measured through attendance rates. Qualitative (open-ended questions) and quantitative data (Likert scales) and an evaluation questionnaire with 10 open-ended questions were collected during the intervention [24].

### Statistical analysis plan

As an acceptability and feasibility pilot, no formal power calculations were conducted. Categorical data were presented as count (%) and compared using chi-squared or Fisher’s exact tests. Numeric data were presented as mean (SD) or median (IQR), depending on distribution. Free-text responses underwent content analysis. Rating and multiple-choice items were analysed descriptively [25]. Analyses of self-report and clinical measures followed four phases:

1. Baseline demographic and clinical characteristics were reported by treatment group to assess randomisation.
2. Descriptive statistics were calculated for primary and secondary outcomes stratified by time and group.
3. Longitudinal changes in the primary outcome (SDQ Impact scale) were analysed with linear mixed models [26], with time and treatment as fixed effects and participants as random effects. This intention-to-treat approach used restricted maximum likelihood to handle missing data.
4. The same models were applied to secondary outcomes.

## Results

**Fig. 1:**
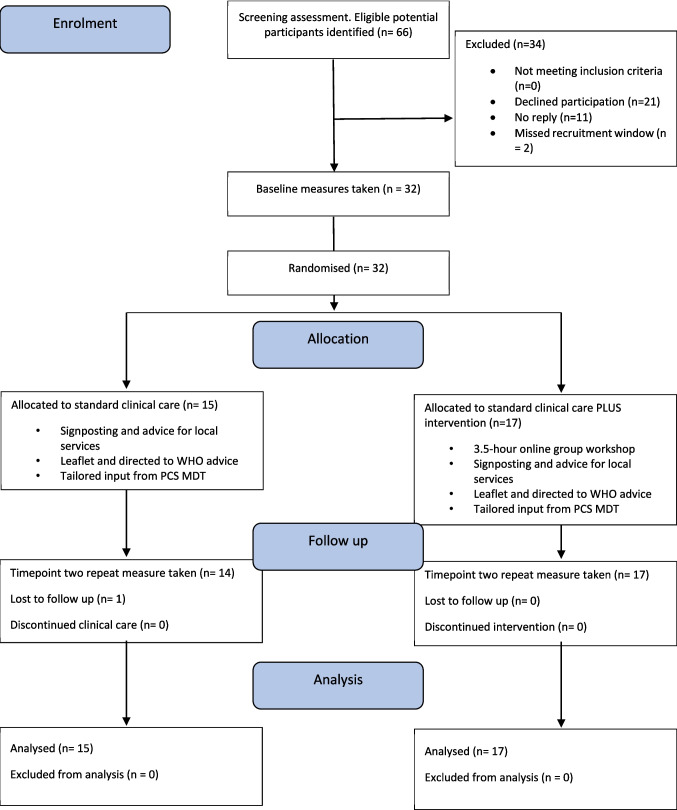
Consort research flow diagram

Sixty-six eligible participants were identified by the clinical teams. Thirty-two consented to take part, twenty-one declined, eleven did not reply and two missed the recruitment window (Fig. 1). Demographic data are reported in Table 1.

**Table 1:** Participant demographic information. Baseline characteristics of the sample, stratified by treatment group

	Control ***N*** = 15	Treatment ***N*** = 17	Total ***N*** = 32			
	*N* (%)	*N* (%)	*N* (%)	*c*	df	*p*
Gender (male)	2 (13.3%)	9 (52.9%)	11 (34.4%)	5.54	1	0.025
Ethnicity (White British)	11 (73.3%)	12 (75.0%)	23 (74.2%)	0.01	1	0.916
Diagnosed with breathing pattern disorder	11 (73.3%)	16 (94%)	27 (84.4%)	2.61	1	0.106
	Mean (SD)	Mean (SD)	Mean (SD)	*t*	df	*p*
Age (years)	15.00 (1.81)	15.58 (1.66)	15.31 (1.73)	− 0.958	30	0.346
Baseline Height (cm)	166.06 (6.44)	169.25 (9.22)	167.76 (8.08)	− 1.12	30	0.272
Baseline Weight (Kg)	60.44 (13.69)	58.37 (12.04)	59.34 (12.67)	0.454	30	0.653
FEV1 (% predicted)	96.68 (11.24)	92.02 (12.5)	94.20 (11.99)	1.101	30	0.280
FEV1 (Z Score)	− 0.27 (0.94)	− 0.71 (1.06)	− 0.50 (1.01)	1.195	29	0.242
Gas Transfer (TLCO SB %predicted)	93.92 (14.66)	94.54 (13.17)	94.26 (13.63)	− 0.124	29	0.902
Multiple Deprivation Rank	15,381.53 (10,217.87)	19,372.17 (7628.50)	17,501.56 (9015.54)	− 1.261	30	0.217

Control and intervention groups were balanced in age, weight, and height. There were more males in the treatment group. Spirometry and gas transfer testing across the cohort were within normal physiological ranges and were not significantly different between groups (Table 1). There was a high rate of diagnosis of BrPD in both groups at baseline (84.4%).

### Symptom description

**Fig. 2:**
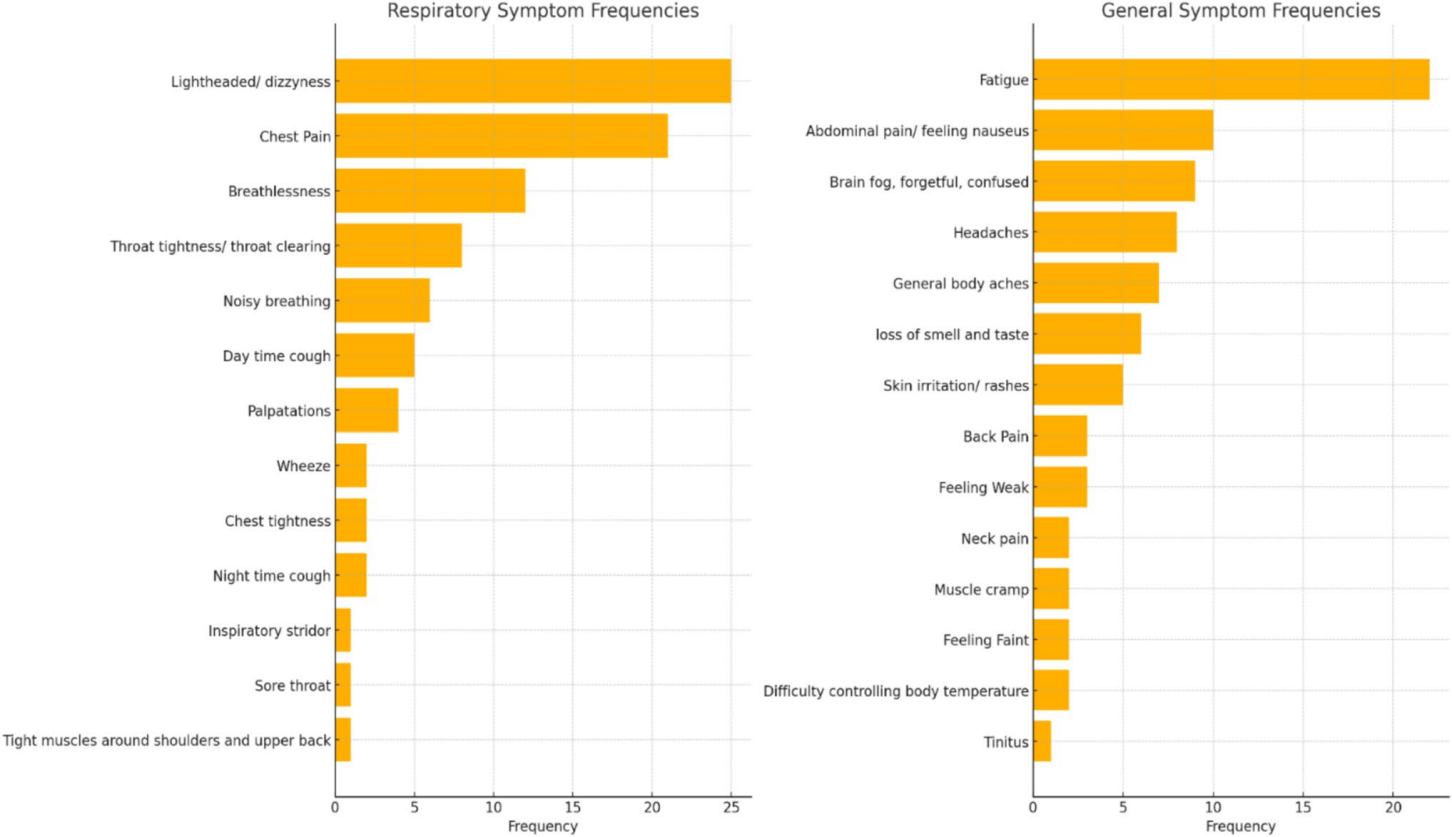
Frequency of symptom presentation for participants at baseline assessment

**Fig. 3:**
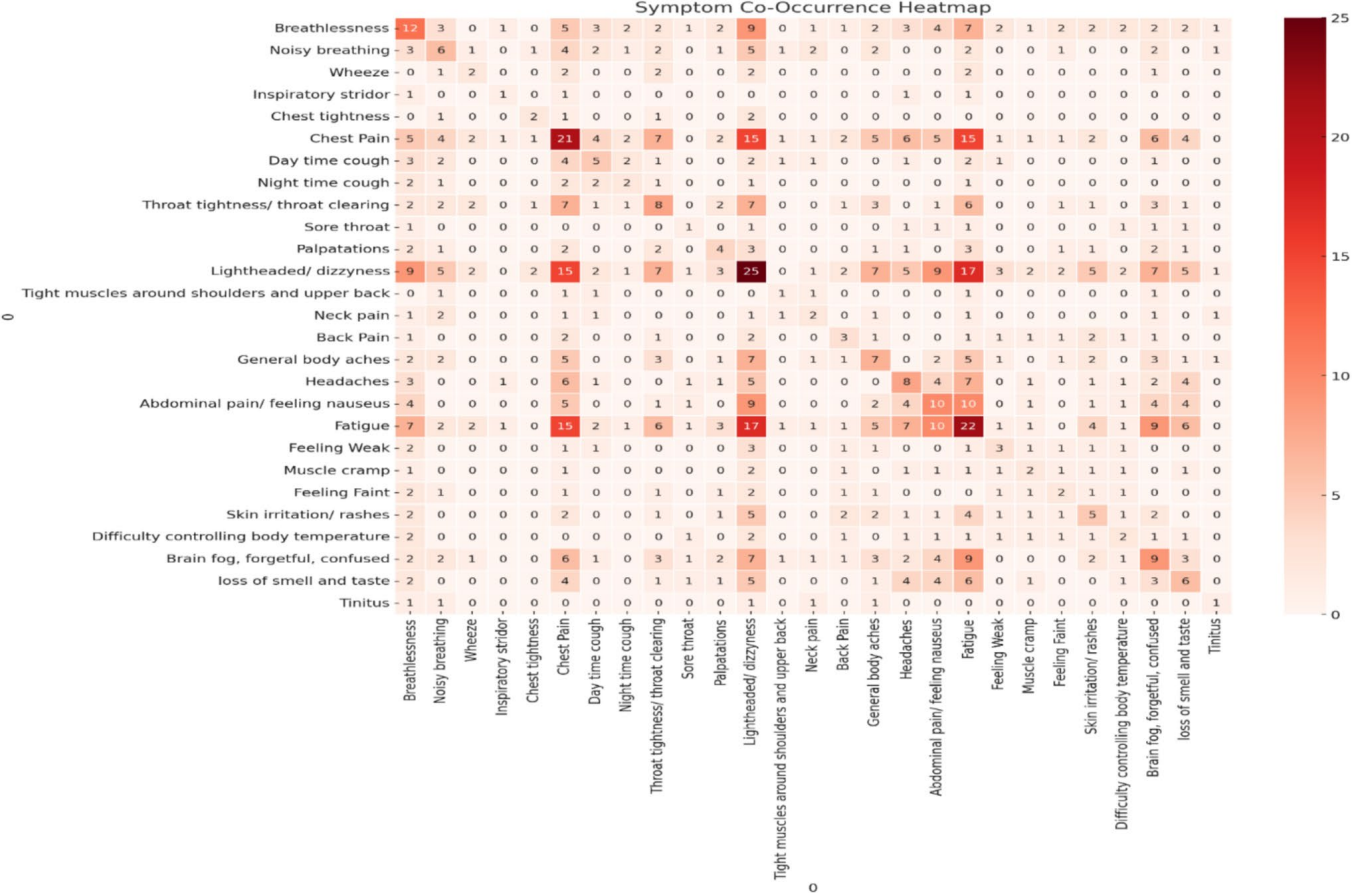
Heatmap of the combination of presenting symptoms for participants at baseline assessment

Symptoms documented at baseline highlighted a wide range of presentations. The reported symptom frequency (Fig. 2) and combination of symptoms were mapped (Fig. 3). The most common combinations of symptoms were light-headedness, fatigue, and chest pain.

### Intervention acceptability and feasibility

#### Feasibility

17/17 young people attended one of four possible online workshops. Two did not complete the session (one developed a headache and one struggled with the group environment). 14/17 (82%) completed the post-session questionnaire.

#### Acceptability

Median rating for the online delivery was 8 (range 5–10). The online format was appreciated for convenience and making participation manageable despite health challenges.

“*It meant I got to do it in a comfortable environment which is familiar to me*.”

Median rating for usefulness was 7 (range 5 to 9), sharing their ideas was 7.5 (range 5 to 10) and would recommend the group was 8 (range 5 to 10). Suggestions included the following:

- “Longer break periods”
- “People at the same stage and ability should be in a group together”
- “Split into shorter meetings like once a week”
- They also wanted more discussions on recovery, coping strategies, or personal stories

While all participants had previously provided formal trial consent/assent covering data collection and anonymised publication of results, a post-intervention questionnaire included a final question confirming that they were happy for their feedback about the intervention to be analysed and directly quoted. Content analysis was used to extract emerging themes. The responses were read by a clinical psychologist (DC). Initial ideas/patterns were noted with words, phrases, or sections coded into potential themes capturing broader patterns or ideas present in the data. OpenAI GPT-4o mini was used to cross-check the coding process as well as the accuracy and consistency of identified themes. The themes were refined by merging, splitting, or discarding those that were vague or overlapping. Three main themes emerged: connecting and sharing, learning from others, calmness and relaxation.

#### Connecting and sharing

Young people valued hearing others’ experiences and connecting with similar struggles, helping foster a sense of community and belonging.

*“Learning that people are in a similar situation helped me not feel alone.”*

*“It’s great to hear from people who are dealing with the same struggles as me.”*

Stories of resilience boosted hope and offered emotional support, increased confidence, and practical learning:

*“Seeing others’ progress made me feel more positive.”*

#### Learning from others

They appreciated the opportunity to share and receive advice:

*“Very helpful to get advice from others.”*

*“It helped me learn how it affects everyone differently.”*

#### Calmness and relaxation

The group was described as a calming environment and a safe space for open discussion. The breathing exercises were described as helpful.

#### Standard clinical treatment

Referrers attend a virtual MDT discussion and receive specialist advice and local signposting. The more severely affected group are accepted into the pan-London PCS services and offered a face-to-face MDT assessment, directed to online patient information (World Health Organisation Long COVID rehabilitation guide (Rehabilitation: self-management of Long COVID for adolescents) and are offered virtual educational sessions on sleep, activity management, and emotional wellbeing. Letters of advice and recommendations for reasonable adjustments are provided for education settings. Some also receive individualised interventions from physiotherapy, occupational therapy, dietetics, and psychological services.

27/32 had at least one additional consultation with the MDT during the research timeframe. All CYP diagnosed with breathing difficulties were referred for physiotherapy. 31/32 had at least 1 additional consultation with a physiotherapist and/or an occupational therapist (mode: 4—range 1–9). 16/32 were offered energy and activity management and 12/32 were seen by psychology services. School liaison was offered to 15/32 young people. It was not possible to record how many young people utilised the digital resources or assess the patient leaflets’ usefulness.

### Intervention effect

Table 2 shows the descriptive statistics for the primary and secondary outcomes stratified by treatment group and time. There was a decrease in the mean SDQ impact score from baseline to post-treatment for both the treatment and control groups. The difference was larger for the treatment (mean *D* = 1.17) than the control group (mean *D* = 0.92), although both differences are small.

**Table 2:** Mean scores of primary and secondary outcome measures by treatment and time

Primary outcome measure	Control		Intervention	
	*n*	Mean (SD)	*n*	Mean (SD)
Baseline Strengths and Difficulties Questionnaire, Impact Score	10	3.30 (2.86)	15	3.46 (3.04)
Timepoint 2 Strengths and Difficulties Questionnaire, Impact Score	13	2.38 (2.32)	17	2.29 (2.33)
Secondary outcome measures				
Baseline Wellness Score (0–100%)	12	43.41 (21.72)	15	34.00 (17.13)
Timepoint 2 Wellness Score (0–100%)	13	60.38 (22.49)	17	60.88 (17.34)
Baseline EQ-5D-Y Total Score	12	10.16 (2.36)	15	10.40 (1.76)
Timepoint 2 EQ-5D-Y Total Score	13	8.30 (1.54)	17	8.58 (1.90)
Baseline 11-item Chandler Fatigue Score (0–33)	8	23.87 (5.22)	12	25.16 (4.56)
Timepoint 2 11-item Chandler Fatigue Score (0–33)	13	18.30 (7.97)	17	17.11 (6.05)
Baseline Nijmegen Questionnaire score (0–64)	15	34.80 (8.58)	17	32.05 (10.57)
Timepoint 2 Nijmegen Questionnaire score (0–64)	13	25.07 (8.45)	17	26.00 (8.33)
Baseline CPET PeakVO_2_ (ml/min)	15	73.40 (15.23)	17	78.11 (22.23)
Timepoint 2 CPET PeakVO_2_ (ml/min)	13	77.46 (11.52)	17	80.64 (18.46)
Baseline CPET Anaerobic threshold (VT2 VO_2_/kg)	13	62.05 (11.80)	13	70.15 (12.69)
Timepoint 2 CPET Anaerobic threshold (VT2 VO_2_/kg)	13	56.10 (15.28)	15	63.22 (11.75)

For secondary outcomes there was a similar pattern with no difference between groups on wellness, EQ5D, CFQ-11, Nijmegen, and anaerobic threshold. This is demonstrated in the linear mixed models results (Table 3).

**Table 3:** Fixed effect omnibus tests for primary and secondary outcome variables

		Treatment	Time	Time × treatment
SDQ Impact Score	f	0.105	1.507	0.230
	Num df	1	1	1
	Den df	30.7	29.5	29.5
	p	0.748	0.229	0.635
Wellness Score percentage (0–100%)	f	0.472	25.381	1.813
	Num df	1	1	1
	Den df	0.472	25.381	1813
	p	0.498	<.001	0.191
EQ-5D-Y total score	f	0.274	16.227	0.008
	Num df	1	1	1
	Den df	30.3	28.6	28.6
	p	0.604	<.001	0.928
11-item Chandler Fatigue Scale (0–33)	f	.001	14.011	0.465
	Num df	1	1	1
	Den df	46.0	46.0	46.0
	p	0.978	<.001	0.499
VT2 VO_2_/kg threshold	f	0.3476	0.5295	0.0418
	Num df	1	1	1
	Den df	29.1	28.4	28.4
	p	0.560	0.473	0.839
Nijmegen questionnaire (0–64)	f	0.0302	28.4345	1.2908
	Num df	1	1	1
	Den df	29.3	28.6	28.6
	p	0.8630	<.001	0.265
Anaerobic threshold VT2 VO_2_/kg	f	3.10	6.40	.000
	Num df	1	1	1
	Den df	28.5	25.4	25.4
	p	0.089	0.018	0.996

There were significant effects over time for Wellness, EQ-5D-Y, CFQ-11, Nijmegen questionnaire, and anaerobic threshold scores. However, none of the interactions were statistically significant, indicating that there were no variables where the change in baseline to post-treatment differed depending on treatment group.

## Discussion

It was feasible to deliver 4 groups over the 6-month intervention period and 32/66 eligible young people were recruited. 15/17 CYP completed a group and feedback suggested the online intervention was acceptable. Young people felt understood, learned practical coping strategies, and reported feeling connected and supported.

At baseline, symptom presentation supports existing literature with fatigue as the dominant symptom. The symptom profile highlights higher rates of light-headedness (25%) and chest pain (21%) compared to other studies [27]. CYP seen in specialist clinics have multiple symptoms, with a significant impact seen on their daily functioning and school attendance [28]. This cohort is likely to have a more severe presentation than those managed outside of the service [29] and the study population may represent a more severely affected group than reported in population-based studies.

Despite breathlessness being the third most common symptom, all 32 CYP had normal spirometry and gas transfer studies. BrPD were identified at rest via physiotherapy assessment in 27 (84%) and via CPET in 29 (91%) at rest and 24 (75%) during exercise within baseline assessments. BrPD diagnosis was agreed in 26 (82%) participants via both assessments. Dizziness, chest pain and fatigue were seen as the most common individual symptoms and commonly occurring combination of symptoms. These symptoms are also seen in people with BrPD and are included in the Nijmegen questionnaire. 24 (75%) participants received physiotherapy clinic sessions for breathing retraining and symptoms within the Nijmegen scores significantly improved over time (P < 0.001). This presentation rate has also been seen in a Swedish study which found BrPD in 95% of respiratory adult clinic attendees, most commonly apical breathing with minimal abdominal movement [30] with 67% reporting sternocostal or intercostal pain. Reported symptoms had poor correlation with spirometry, muscle strength or thoracic mobility, suggesting respiratory symptom assessment to go beyond respiratory spirometry. This is the first time this has been reported in the paediatric population.

The results show that there was no standard treatment offered to both control and intervention groups. Following the initial MDT assessment, several additional assessments and interventions were offered including MDT follow-up with 2–5 professionals and interventions that included energy/pacing management, exercise prescription, posture/MSK interventions, school liaison, breathing retraining, relaxation techniques, nutrition review and individualised psychological support and rehabilitation for the young person and/or their family. Five different onward referral pathways to local services were identified (consultant only, occupational therapy, physiotherapy, child and adolescent mental health services (CAMHS)/psychology, psychiatrist, and social workers).

## Limitations

A limitation of this study relates to the size of the eligible population invited to participate relative to the wider number of children and young people living with post-COVID syndrome in the UK. Although 66 young people were identified as potentially suitable for the intervention across three specialist centres, this number reflects pragmatic constraints of the study design rather than population availability. The project involved a time-limited, partially funded programme of work that included co-design of the intervention, site set-up across three centres, delivery of the intervention, and a longitudinal follow-up period of six months. As a result, the recruitment window was necessarily restricted by the time required to complete each phase of the research.

Importantly, the modest recruitment numbers should not be interpreted as indicating a lack of need for such interventions beyond the participating centres. Rather, they highlight the challenges of conducting co-produced, interdisciplinary research within fixed timeframes and service contexts. Future research would benefit from larger-scale, multi-centre implementation studies to explore demand, reach, and scalability of this intervention across a broader range of services and geographical settings.

The study was not powered to detect change over time. The lack of a clearer standard treatment also made it difficult to identify which input was potentially effective. The complexity and intensity of the input being received by the controls made it very unlikely that an impact from the intervention would be detected. The small numbers limited the potential analysis of individual differences and whether they may have had a differential impact on outcome, e.g. premorbid psychiatric comorbidity and/or neurodevelopmental difference. Given the high prevalence of both in the PCS population, this has the potential to guide clinicians in terms of potential adaptations.

The primary outcome measure (SDQ impact score) was chosen before the intervention had been co-designed. As the intervention was designed to have an impact on attitude to illness and recovery as well as symptom changes, a primary outcome measure to assess this, like school attendance and inclusion in life activities, would have better evaluated its effectiveness.

A consideration for this study includes the lack of blinding due to the position of the lead researcher who was the clinical specialist physiotherapist in the intervention as well as the breathing pattern specialist clinic lead. Ideally, funding would have allowed for more research staff to be able to separate the research and clinical element of the study.

A significant percentage of young people interested in participating in the study struggled with symptoms of breathlessness. As these investigations are not part of the usual tests, there could be a potential recruitment bias. Once we had identified BrPD in the participants, the research team decided to respond to the clinical need, which in this instance was to offer breathing retraining clinic appointments, potentially reducing the chance of the intervention having an impact.

Future learning from conducting this study also includes the need to explore parental perception of health change, which can be important in complex conditions such as PCS. There was suspected reporter bias in some of the baseline assessment questionnaires with uninvited parental influence.

The intervention materials were made available to teams following completion of the study. Delivery of the intervention requires the involvement of one psychologist and one physiotherapist, and services should consider how best to balance group-based delivery with the need for timely, individualised care for adolescents and young people. While no specific benefit was demonstrated within this study, implementation may be most appropriate in settings where sufficient multidisciplinary resources are available to support young people’s broader clinical needs.

## Conclusions

CYP with PCS present with a complex range of physical, psychological and functional symptoms that need holistic management; further studies are needed to determine the trajectory of improvements seen after six months. Learning from this study identified the need to include measures that are reflective of what is happening in the group; for example, measures of social connectiveness and adherence to breathing exercises.

Breathlessness, chest pain, and fatigue are highly prevalent in this population with a high incidence rate of BrPD. Exploration of the effect of breathing pattern retraining over time would be important to review the effect of these interventions.

## Supplementary Information

Below is the link to the electronic supplementary material.

ESM 1 Supplementary Material 1 (DOCX 18.8 KB)

## Data Availability

No datasets were generated or analysed during the current study.

## Abbreviations

BrPD: Breathing pattern dysfunctions/disorders
CAMHS: Child and Adolescent Mental Health Services
CFQ-: 11-Item Chalder Fatigue Questionnaire
CPET: Cardiopulmonary Exercise Test
CYP: Children and young people
ISARIC: International Severe Acute Respiratory and emerging Infection Consortium
MDT: Multidisciplinary team
PCS: Post COVID syndrome
SDQ: Strengths and Difficulties Questionnaire
