# Supplementary material for: The breath and mind connection in young people with post-COVID syndrome: feasibility and acceptability of a pilot randomised co-designed intervention
Source: Eur J Pediatr. 2026 Mar 16;185(4):185. doi: 10.1007/s00431-026-06840-7 (PMC12992349; doi:10.1007/s00431-026-06840-7)
Supplement: Supplementary file 1 — Supplementary Material 1 (DOCX 18.8 KB) [file 431_2026_6840_MOESM1_ESM.docx]

Supplementary materials

Overview of journey of life sections for the intervention group session

| Journey of life sections | What it represents | Intention |
| --- | --- | --- |
| Journey | Introducing life as a journey | Housekeeping, setting intention and reviewing expectations for the session and introduction to life as a journey.  Review of their place on their journey and reflection on how they are feeling. |
| Breathing exercise 1: Conscious breathing practice: Introduction to healthy breathing, overlap with symptoms of dysfunctional breathing with PCS | | |
| Energy Bar | Activities they enjoy and skills | Identifying skills and abilities they have and what they take with them on the journey |
| Compass | Key values, beliefs, and principles | Key values, beliefs and principles to guide them through difficult times. |
| Breathing exercise 2: Energising breath exercise to invigorate and motivate | | |
| Joining together |  |  |
| Obstacles | Challenges for all young people  Additional challenges for young people with PCS | Prepare thinking about obstacles as a collective challenge for all young people.  Reflect on additional challenges for young people with PCS |
| Breath exercise 3: Calming breath – to help avoid fight or flight response and navigate panic | | |
| Camera | Skills and abilities that made these achievements possible | Tips, Tricks and Hacks to identify how they achieved success |
| Keys | Success, achievements | Highlight key successes they have achieved and what has gone well on their journey so far |
| Travel companions | Appreciating important people in our lives | Identify who has helped them along the way |
| Map | Hopes, dreams, and wishes for the future | What will they be doing 6 months from now? |
|  |  |  |
| Raincoats | Strategies for living with PCS/ things that keep us going. | Tips, Tricks and hacks for what keeps them going |
| Message in the bottle | Sharing a message to other young people who are starting this journey | What would they like to say to someone else starting their long-covid journey? What advice would they like to pass on? |
| Breathing exercise 4: Peaceful, resilient and grateful breath to allow for mindfulness and reflection. | | |
|  |  | Final review of their place on their journey and reflection on how they are feeling. |

**CPET protocol and analysis**

Incremental CPET used Bruce protocol on a treadmill or ramp protocol on a cycle ergometer to volitional fatigue. Maximal effort required RER>1.1 and peak HR>85% predicted. Exercise capacity limitation was VO2max<85% predicted. Reduced VO2max with normal ventilatory reserve suggested deconditioning. Impaired respiratory response was VO2max<85% with ventilatory reserve<15%.

Reduced VO2max, early anaerobic threshold, no cardiac reserve and plenty of ventilatory reserve suggested physical deconditioning. Impaired respiratory response to exercise was defined as reduced VO2max<85% with ventilatory reserve<15%. Cardiovascular limitation was defined as a reduced VO2max<85% with impaired O2 pulse (VO2/HR), early anaerobic threshold (VT2) <40%, low peak heart rate and normal ventilatory reserve.

Duration of the test, reason for stopping, BORG breathlessness scales pre and post-test were documented. Details on physiological measurements at the peak work rate include, load, heart rate, breathing frequency, oxygen saturations. Physiological measures documented include VO2 max and anaerobic threshold.
